# The Effects of Low-Level Laser Therapy, 670 nm, on Epiphyseal Growth in Rats

**DOI:** 10.1100/2012/231723

**Published:** 2012-04-30

**Authors:** Adriana Regina de Andrade, Anamaria Meireles, Elisangela Lourdes Artifon, Rose Meire Costa Brancalhão, José Roberto Leonel Ferreira, Gladson Ricardo Flor Bertolini

**Affiliations:** Laboratory of the Study of Injuries and Physiotherapy Resources, State University of Western Paraná (UNIOESTE), Rua Universitária 2069, Jd. Universitário, P.O. Box 711, 85819-110 Cascavel, PR, Brazil

## Abstract

The longitudinal growth of long bones is attributed to epiphyseal growth. However, the effects of low-level laser therapy (LLLT) in such structures has still not been studied extensively in the literature. Therefore, the aim of this study was to evaluate the use of LLLT, 670 nm, at three different doses on the epiphyseal growth of the right tibia of rats. Twenty-one Wistar rats, aged four weeks, were subjected to the application of LLLT, with dosage according to the group (G4: were submitted to the application of 4 J/cm^2^; G8: were submitted to the application of 8 J/cm^2^; G16: were submitted to the application of 16 J/cm^2^). After completion of protocol they were kept until they were 14 weeks of age and then submitted to a radiological examination (evaluation of limb length) and euthanised. The histological analysis of the growth plates (total thickness and hypertrophic and proliferative zones) was then performed. Comparisons were made with the untreated left tibia. No differences were observed in any of the reviews (radiological and histological), when comparing the right sides (treated) to the left (untreated). It was concluded that the treatment with LLLT within the parameters used caused changes neither in areas of the epiphyseal cartilage nor in the final length of limbs.

## 1. Introduction

 The longitudinal growth of long bones is assigned to a structure called the growth plate, physis [1], epiphyseal growth plate, or epiphyseal plate [2]. This can be divided into zones of chondrocytes, separated by various stages of differentiation: resting, proliferative, hypertrophic, and vascular invasion. Proliferative and hypertrophic zones are mainly responsible for bone growth in length [3].

 The growth plate in children and teenagers is more fragile than the surrounding structures, which predisposes the possibility of damage to it. Disturbances in epiphyseal growth due to injuries can result in limb length discrepancy, angular deformity, or alteration of mechanical joints, causing significant disability [2]. The main causes of injuries in the growth plate are acute traumatic [1], such as the traumatic dislocation of the proximal radial epiphysis [4]. Other disorders that can involve the physis are infections and tumors [1], in addition to repetitive stress injuries, which can cause irreversible damage to growing bones [5].

 Physiotherapy offers resources such as therapeutic ultrasound and low level laser therapy (LLLT), which may help in the treatment of these diseases. However, their effects near the growth cartilage are controversial [6–8]. The use of ultrasound is not recommended for pediatric patients. Both the thermal [9], and nonthermal effects of ultrasound can produce changes in epiphyseal growth [10]. Nonthermal effects occur through the alteration of membrane permeability to calcium influx, modulating the nuclear proliferation and also the RNA transduce [11]. These effects are also found in low level laser therapy [12].

 The effect produced by LLLT irradiation is due to the absorption of energy by specific photoreceptors, such as porphyrin and cytochrome c oxidase. This generates increased production of oxygen, which stimulates the activity of mitochondria in ATP production, increases chemiosmosis, the production of DNA and the influx of calcium into the cytoplasm, which leads to mitosis and cell proliferation [13]. These effects cause increased cell proliferation and migration, increased tissue oxygenation, and the modulation of cytokine levels, growth factors, and inflammatory mediators [14]. As a result of these reactions, the laser produces anti-inflammatory and analgesic effects, promotes healing, the formation of blood vessels, and the stimulation of fibroblasts and bone cells [15].

 Studies on the effects of LLLT in epiphyseal growth are few and controversial. Cheetham et al. [7] report that the use of LLLT caused no significant effect on the growth plates in the knees of rats when histomorphometric analysis was performed. However, Cressoni et al. [8] found changes in the thickness of the epiphyseal cartilage and increase in the number of chondrocytes, but no change in bone length.

 Due to the small number of studies and conflicting results regarding the effects of LLLT on epiphyseal growth further studies are necessary to prove whether low-level laser treatment can be used near the epiphyseal cartilage. The aim of this study is to evaluate the use of LLLT, 670 nm in three different doses on the tibia growth plate of Wistar rats by histologic analysis and to check for changes in the tibia by means of radiography.

## 2. Methods

### 2.1. Sample and Study Characteristics

 The present study was characterised as a quantitative, experimental study. It was approved by the Ethics Committee on Animal Experiments and Practical Studies of the West of Paraná State University (UNIOESTE) under Protocol number 0910.

Twenty-one male Wistar rats aged four weeks were used. These animals had not reached skeletal maturity [16] and weighed an average of 137.42 ± 9.72 grams. The animals were obtained from the Unioeste Central Vivarium and were kept in the vivarium sector in polypropylene cages with three or four animals in each cage. These rats were fed with water and food *ad libitum*, remaining under controlled conditions with a temperature of  23 ± 2°C and a light/dark cycle controlled every 12 hours.

These animals were submitted to a protocol of application of LLLT. After the completion of this protocol they were kept in the vivarium until they were fourteen weeks old, in other words bone maturity had been reached [16]. The animals then underwent a radiologic examination to measure the length of limbs and/or any epiphyseal changes. The day after the radiological examination the animals were euthanised by decapitation in order to perform the histological analysis of the epiphyseal growth.

 The sample was divided randomly into three groups:

- G4 (*n* = 7): submitted to the application of 4 J/cm^2^;
- G8 (*n* = 7): submitted to the application of 8 J/cm^2^;
- G16 (*n* = 7): submitted to the application of 16 J/cm^2^.

### 2.2. Laser Stimulation Protocol

 The region where the LLLT was applied had been previously shaved when the animals were four weeks old and they were manually restrained during application. The LLLT transmitter was positioned at 90 degrees to the treated site. The laser was applied to only one point in the medial epiphyseal growth region of the right tibia, the left limb being the control side. The applications were performed for 10 consecutive days. The equipment used was of 30 mW and 670 nm wavelengths. The emission form was continuous and the irradiation area was 0.06410 cm^2^. The power was previously measured.

### 2.3. Radiological Examination

 For the radiological exam, a computerised radiology system was used (CR). The animals were anesthetised with ketamine (95 mg/kg) and xylazine (12 mg/kg) intraperitoneally, before being radiographed. The parameters used were Buck X-ray table, 6.6 mAs, 50 kV, 100 mA focus, exposure time of 0.066 ms, anterior, and posterior incidence. The animals were X-rayed two by two and these parameters had been tested previously.

After the images were obtained they were archived in the working section of the radiology equipment and then subjected to special techniques and filters for bone structures in order to obtain the best resolution of these images. Using a digital radiology program, limb length measurements were taken from these images. For this, a line was drawn from the midpoint of the proximal tibia to the midpoint of the distal tibia and this length was measured. The X-ray analysis was performed by a radiologist who is a member of the Brazilian College of Radiology.

### 2.4. Histological Analysis

 After the animals were euthanised, the tibias were disarticulated at the knees and ankles and the soft tissues were removed. The tibias were kept in 7% formalin for three days to fix. Later they were placed in a solution of 10% nitric acid for 30 days, for decalcification.

At the end of this period the material received the following histological processing: dehydration, diaphanisation, paraffin embedding, and microtomy, coronal sections were performed to a thickness of 7 *μ*m. Two histological slides for each epiphyseal growth were prepared which were stained with hematoxylin-eosin (HE) and only one slide was examined for epiphysis.

To analyse these slides, we used a camera attached to a microscope and an image program. We took three images of each slide: medial, intermediate, and lateral regions of the epiphyseal growth. From these images, measurements were made of the epiphyseal growth, total thickness, and the hypertrophic and proliferative layers. Five measurements were performed for each evaluated region, making a total of 45 measurements for each slide.

### 2.5. Statistical Analysis

 For comparison of the right side with the left side we used the paired *t*-test. For the different regions of the epiphysis ANOVA was used repeated with Bonferroni posttest. The level of significance was *P* < 0.05.

## 3. Results

### 3.1. Radiographic Analysis

 When the treated limb was compared with the untreated limb there was no significant difference between the length of the limbs (G4—*P* = 0,0881; G8—*P* = 0,0892; G16—*P* = 0,2308) in all groups.

**Figure 1:**
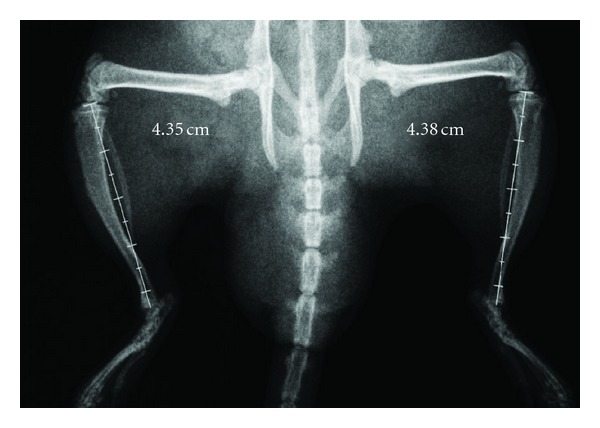
Radiography of an animal from G4. Measurement of limb length.

 With respect to radiographic analysis, the medical report showed: soft tissue unchanged; bone texture preserved; joint surfaces smooth, and regular and symmetrical epiphyseal nucleus with normal dimensions (Figure 1).

### 3.2. Histological Analysis

**Figure 2:**
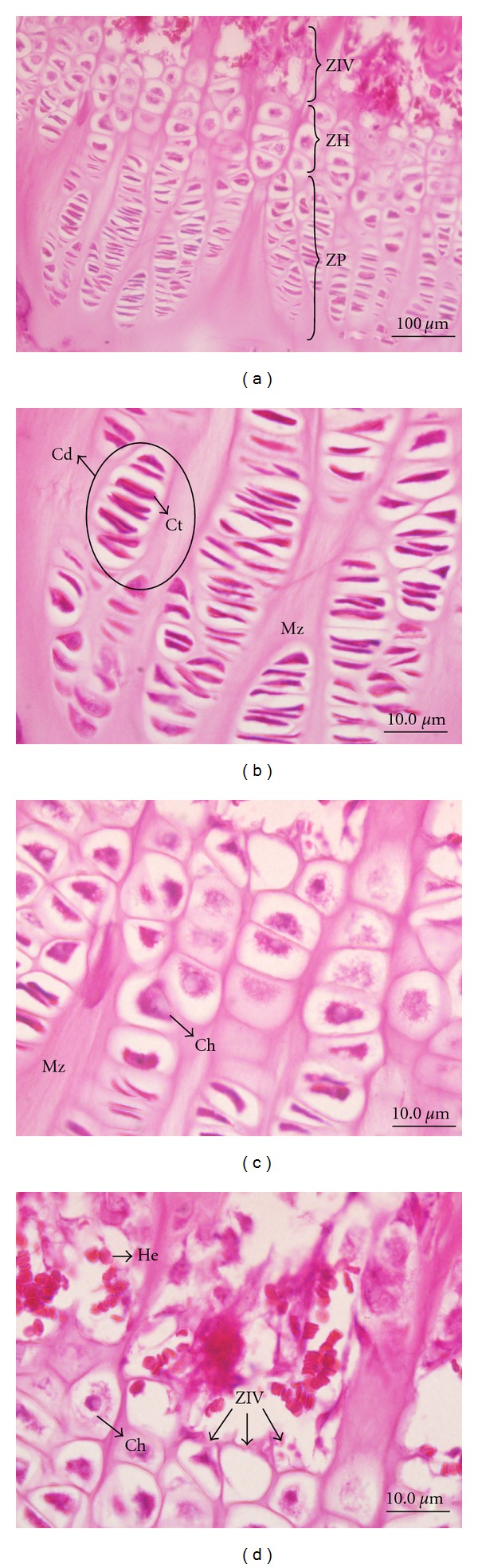
Photomicrographs of Wistar rats' tibia, showing the epiphyseal cartilage in the coronal plane. Stained by hematoxylin and eosin. In image (a), areas of the epiphyseal disc: proliferative (ZP), hypertrophic (ZH), and vascular invasion (ZIV). These zones are seen in details in the images in (b), (c) and (d). Image (b) shows the ZP, where the chondrocytes are seen flattened into columns (Ct) in condron (Cd) and cartilage matrix (Mz). In image (c), ZH and in image (d), ZIV, hypertrophic chondrocytes (Ch), and erythrocytes (He).

 In the histological analysis of the epiphyseal cartilage zones, there was no significant difference when comparing medial, lateral, and intermediate regions of the epiphysis, regarding the total thickness and proliferative and hypertrophic zones (*P* > 0.05) (Figure 2). Comparing the treated side with the untreated, no significant difference was found in all groups with respect to the total thickness (G4—*P* = 0.3973; G8—*P* = 0.7830; G16—*P* = 0.6888), and the hypertrophic zones (G4—*P* = 0.5673; G8—*P* = 0.8717; G16—*P* = 0.3417) and proliferative zones (G4—*P* = 0.3121; G8—*P* = 4161; G16—*P* = 0.7837) of the epiphysis.

## 4. Discussion

 In this study, we evaluated the possible effect of LLLT on epiphyseal growth through both radiological and histomorphometric analysis. The radiological analysis showed that application of low level LLLT, 670 nm, produced no difference between the length of treated and untreated limbs. Also, in histological analysis no change was observed in total thickness and in the proliferative and hypertrophic zones of the different regions of the epiphyseal growth. Likewise, no histological difference was detected between the treated side and the control.

 Unlike our study, Seifi et al. [17] applied LLLT with a wavelength of 904 nm, in the condyles of 42-day-old rats with 0.24 J/cm^2^, for prolonged periods. They observed a significant increase in the animals' mandible length. They report that this increase may be due to the increased formation of bone and cartilage due to the stimulating mechanisms of laser therapy.

Similar to our study, Cressoni et al. [8] evaluated the growth plate of rats using histological and radiographic analysis after they had been submitted to the application of LLLT with a wavelength of 830 nm, and doses of 5 J/cm^2^ and 15 J/cm^2^. They observed that the LLLT produced no change in bone length, supporting the present research. However, it was observed that the animals submitted to a 15 J/cm^2^ dose showed lower thickness of the hypertrophic layer of the epiphysis. According to the authors, this may be related to a possible acceleration of the ossification stage. However, this effect might have not occurred in our study, since there was no thinning of the hypertrophic zone.

 Cheetham et al. [7] applied LLLT with a wavelength of 820 nm and energy density of 5 J/cm^2^ in the growth plate of young female rats. As in the present study, no significant effect was found on the treated epiphysis compared with the untreated ones.

 Morein et al. [18] applied high power laser (CO_2_, 7–10 W) to the epiphyseal plate of rabbits and noted the destruction of the epiphyseal plate. This was replaced by calluses formed by the adjacent bone, causing the cessation of bone growth. It is noteworthy that LLLT has different characteristics from those of high-level laser power and therefore we did not find signs of destruction of the epiphysis in our study.

Longtitudinal bone growth is related to the progression of different stages of the differentiation of chondrocytes. The hypertrophy of these cells is responsible for 44–59% of the growth of long bones and the remainder is due to matrix synthesis and the proliferation of chondrocytes. The factors that stimulate hypertrophy of chondrocytes are very likely to involve changes in ion channels [19]. The laser stimulates the increased synthesis of ATP and the proton gradient, which leads to increased channel activity of Na^+^/H^+^ and Ca^+^/Na^+^ [14]. This effect could stimulate the hypertrophy of chondrocytes. However, it is believed that this did not occur in our study, since there was no change in the epiphyseal growth.

Another factor that stimulates cellular activation and proliferation is the increased concentration of intracellular calcium. It is thought that the laser irradiation stimulates the absorption of a photon by a photoreceptor, followed by changes in plasma membrane and the entry of calcium ions into the cell and subsequent cell activation [20]. Cells from the calvaria of fetal rats and isolated from osteoblasts have been irradiated with LLLT (830 nm), stimulating cell proliferation and the gene expression of osteocalcin [21]. According to Hamblin and Demidova [14], LLLT directly stimulates the regulation of the expression of specific genes or indirectly regulates the expression of genes related to synthesis and DNA repair and cell metabolism.

 It is also noteworthy that the proliferation of chondrocytes is marked by the protein related to the parathyroid hormone, which binds to the surface of the reserve zone chondrocytes, stimulating their proliferation, and also to the proliferative zone chondrocytes by inhibiting their differentiation into hypertrophic chondrocytes; thus ensuring the maintenance of disc growth. This peptide is synthesised by cells of the chondrogenic layer of the perichondrium [22].

With skeletal maturity there is a decreased rate of longitudinal bone growth. This decrease is associated with structural changes in the physis, such as a gradual decrease in the width of the growth plate due to a reduction of the height of the proliferative and hypertrophic layers as well as a reduction in the size of these cells [19]. Therefore, we attempted to analyse the total thickness and the proliferative and hypertrophic zones of the epiphysis, but no differences were presented between the stimulated or unstimulated sides, not even with respect to the medial, intermediate, and lateral regions of the epiphysis, that is, close or distant to the area of stimulation.

 It should be noted as limitations to this study that we only used one wavelength and a narrow spectrum of therapeutic doses. It is suggested that further studies are conducted with different wavelengths and a larger spectrum of doses.

 We concluded that treatment with LLLT, with a wavelength of 670 nm, in the parameters used in this study, did not cause changes in the zones of epiphyseal cartilage as well as the final length of limbs.
